# Cutaneous Metastasis of Gastric Adenocarcinoma: A Report of a Rare Case and a Literature Review

**DOI:** 10.7759/cureus.92805

**Published:** 2025-09-20

**Authors:** Kalan Patel, Daniel Daugherty, Ashwin Jagadish, Shivani Patel, Dharmen Patel, Amani Al-Housseiny, Thomas Soike

**Affiliations:** 1 Internal Medicine, East Tennessee State University James H. Quillen College of Medicine, Johnson City, USA; 2 College of Medicine, East Tennessee State University James H. Quillen College of Medicine, Johnson City, USA; 3 Oncology, Tennessee Cancer Specialists, Johnson City, USA; 4 Pathology, East Tennessee State University James H. Quillen College of Medicine, Johnson City, USA

**Keywords:** cutaneous metastasis, gastric adenocarcinoma, internal medicine, malignancy, oncology

## Abstract

Cutaneous metastases from visceral malignancies occur in a minority of patients. Gastric adenocarcinoma rarely seeds the skin and often masquerades as benign dermatoses, which can delay diagnosis. We report an 80-year-old man with stage IV gastric adenocarcinoma with rapidly enlarging nodules of the lower lip and chin. A pedunculated lesion of the right lower lip involving the vermilion border and a nodule on the left chin had enlarged over five weeks. The lip excision measured 2.6 × 2.1 cm, and the chin excision 1.2 × 2.2 cm. The lip specimen showed cytoplasmic positivity for pan-cytokeratin (AE1/AE3) and a markedly elevated Ki-67 proliferation index. The findings supported metastatic adenocarcinoma consistent with the patient’s known gastric primary. This case highlights that rapidly evolving or indurated skin lesions can occur even at atypical sites such as the lip and chin. Thus, an urgent biopsy in patients with known or suspected malignancy can be beneficial. Histologic and immunophenotypic confirmation enables timely staging, realistic prognostic discussions, and symptom-directed interventions. While local excision can provide meaningful palliation, cutaneous dissemination from gastric adenocarcinoma generally reflects widespread disease and limited survival.

## Introduction

Cutaneous metastasis is an uncommon manifestation of visceral malignancy, reported in only 0.7-9% of all patients with cancer [1]. In the largest clinicopathological series to date, skin involvement was identified in 5% of individuals with disseminated carcinoma, most frequently arising from breast, lung, and colorectal primaries [2]. A subsequent meta-analysis, which pooled more than 20,000 cases, confirmed a similar overall prevalence and underscored the tendency for lesions to appear late in the course of the disease [3]. Occasionally, however, the skin may herald an otherwise occult neoplasm: Lookingbill et al. found that in 0.8% of 7,316 cancer patients, the initial sign of an internal malignancy was a metastatic cutaneous focus [4].

Gastric adenocarcinoma rarely seeds the skin. In the classic autopsy studies of Brownstein and Helwig, only 6 of 4,020 cutaneous deposits (0.15%) originated from the stomach [5]. Subsequent reviews and isolated case reports suggest a contemporary incidence of 0.2-0.8%, usually presenting as a solitary, firm nodule on the abdominal wall-the so-called Sister Mary Joseph nodule-or, even more rarely, as multiple disseminated papules and nodules [6-8]. Since these lesions are often mistaken for benign dermatoses, timely recognition is challenging yet critically important: cutaneous spread signifies advanced disease and portends a median survival measured in months [8-10].

Histopathologically, gastric metastases very often exhibit poorly differentiated signet-ring or mucin-producing adenocarcinoma, mirroring the primary tumor [9]. Their pathogenesis remains incompletely understood but is thought to involve hematogenous dissemination, lymphatic embolization, or direct extension along embryonic ligaments [10]. Regardless of the route, skin involvement often invariably reflects systemic spread and necessitates prompt staging and palliative-oriented therapy.

We report a case of generalized cutaneous metastases arising from gastric adenocarcinoma, highlighting the diagnostic pitfalls, histological features, and prognostic implications of this striking presentation. Our experience reinforces the need for heightened clinical suspicion when evaluating new or unusual skin lesions in patients with known or even suspected gastrointestinal malignancy.

## Case presentation

**Figure 1:**
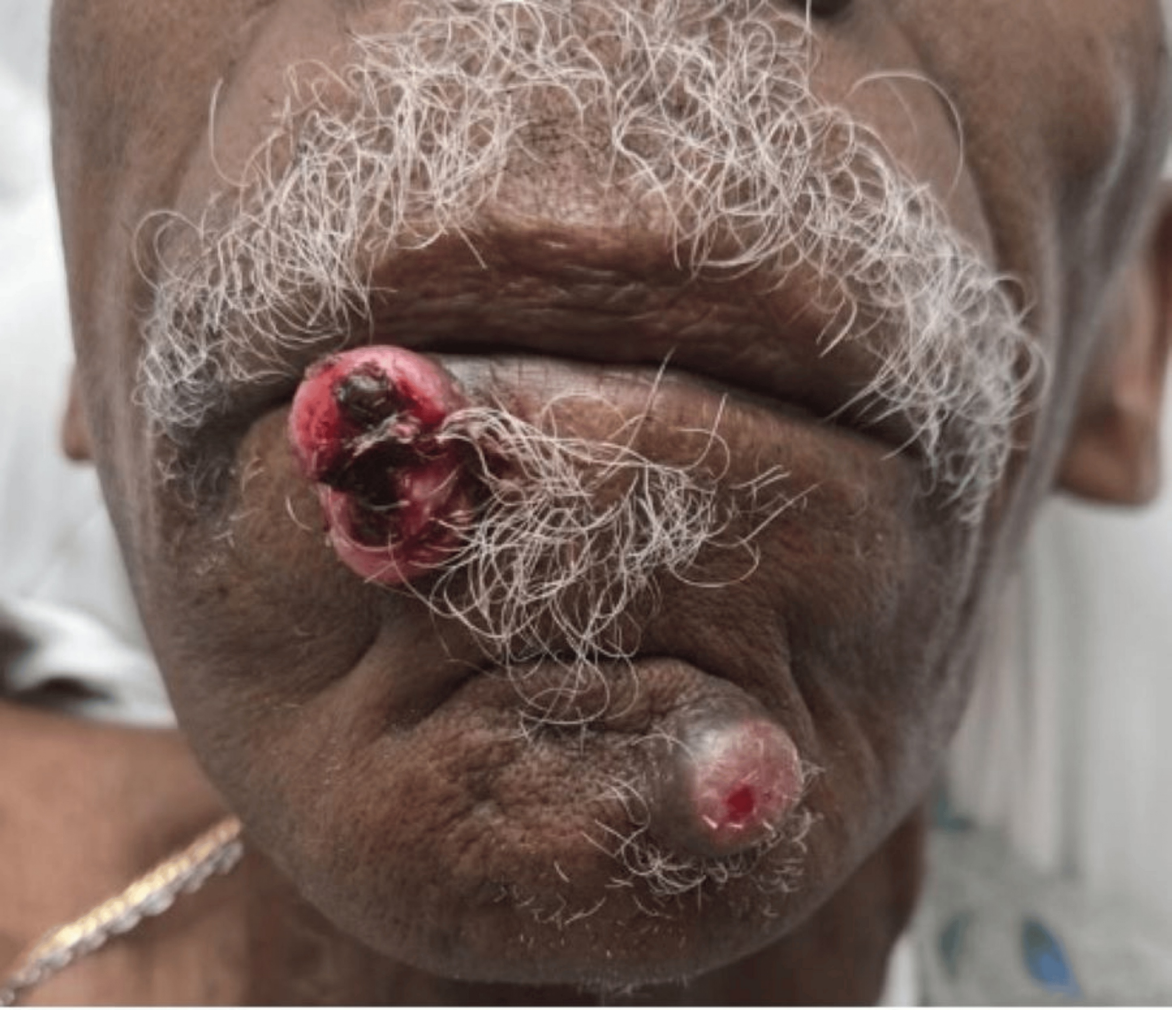
Lip and chin lesions Two rapidly progressive cutaneous lesions on the right lower lip and left chin.

An 80-year-old man with a medical history of stage IV gastric adenocarcinoma diagnosed in 2023, rapidly progressive lower lip and chin lesions, possible metastatic pulmonary nodules, chronic obstructive pulmonary disease with severe emphysema, benign prostatic hyperplasia, gastroesophageal reflux disease, previous alcohol dependence, nicotine dependence, and anemia was referred to the emergency room by the cancer center, where he was being treated for gastric adenocarcinoma, due to low hemoglobin levels. In the emergency department (ED), his blood pressure was 139/88 mmHg, heart rate was 110 beats per minute, and oxygen saturation was 99% on room air. He denied any systemic symptoms of hemoptysis, hematemesis, hematochezia, melena, hematuria, or history of previous need for blood transfusions, but he was cachexic. In addition to his primary concerns, the lower lip and chin lesions were of concern for the patient (Figure 1). In the ED, his hemoglobin was 6.5 g/dl (reference range: 13.5g/dl-17.5g/dl); 1 unit of packed red blood cells (PRBCs) with a 500 mL bolus was given before he was admitted. 

**Figure 2:**
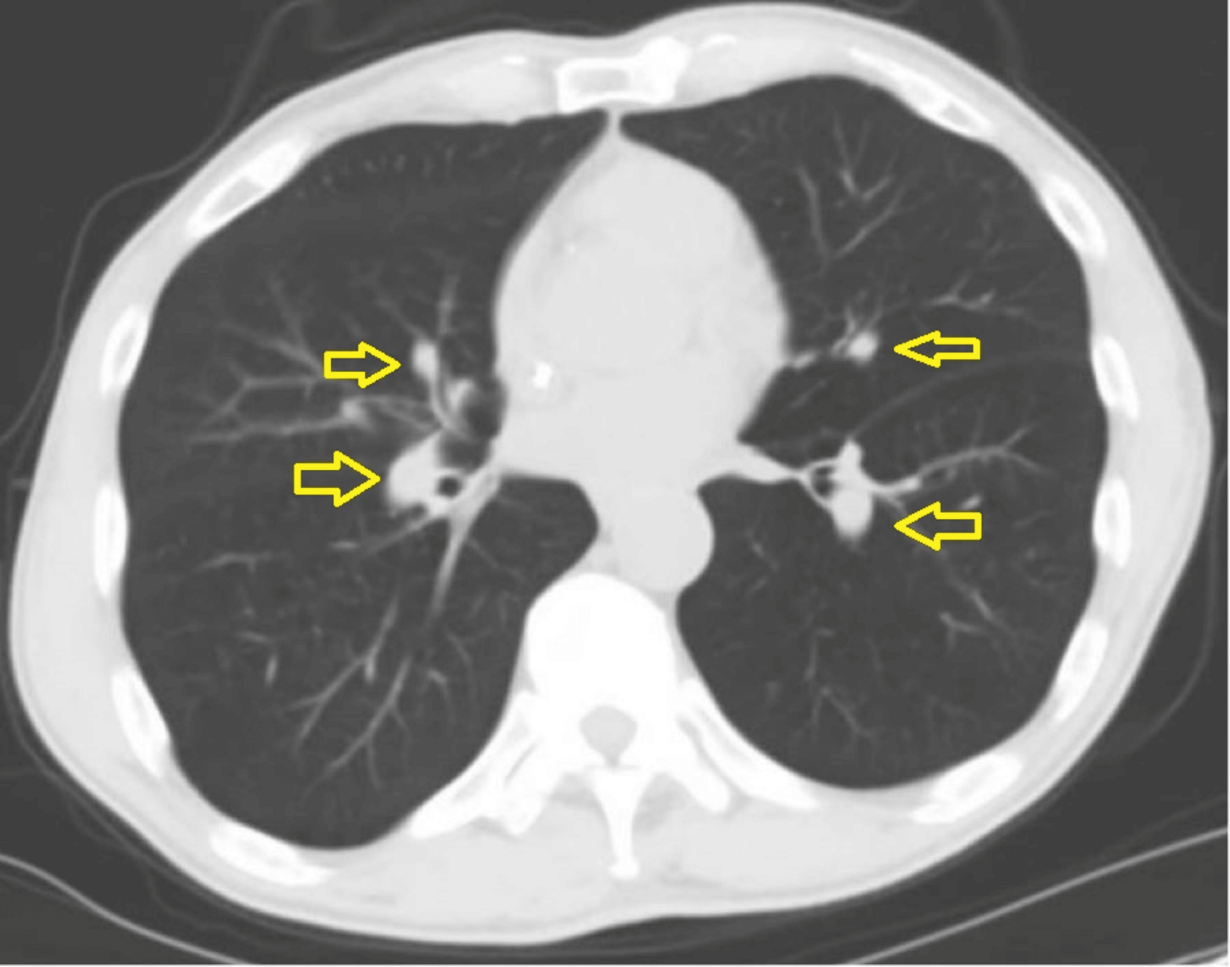
Computed tomography of the chest Arrows indicate the nodules.

Prior to admission, the patient was started on combination therapy of folinic acid, 5-flurouracil, and irinotecan (FORFIRI) to treat his gastric adenocarcinoma after developing an allergic reaction to folinic acid, 5-flurouracil, and oxaliplatin (FOLFOX) in 2024 and concerns of pulmonary toxicity due to possible metastatic pulmonary nodules while on paclitaxel and ramucirumab. Esophagogastroduodenoscopy a month prior indicated known gastric cancer with no new ulcers or bleeding, and a normal esophagus and duodenum. Computed tomography of the chest (Figure 2) indicated enlarging pulmonary nodules suspicious of metastatic disease; biopsy was declined by interventional radiology due to an access issue.

On admission, plastic surgery was consulted for the lip and chin lesions that had progressed rapidly in the past five weeks; one of the lesions had vermilion borders and was pedunculated. Surgical excision of both lesions after a punch biopsy was done for the patient’s comfort. The lip excision measured 2.6 x 2.1 cm, and the chin excision measured 1.2 x 2.2 cm.

**Figure 3:**
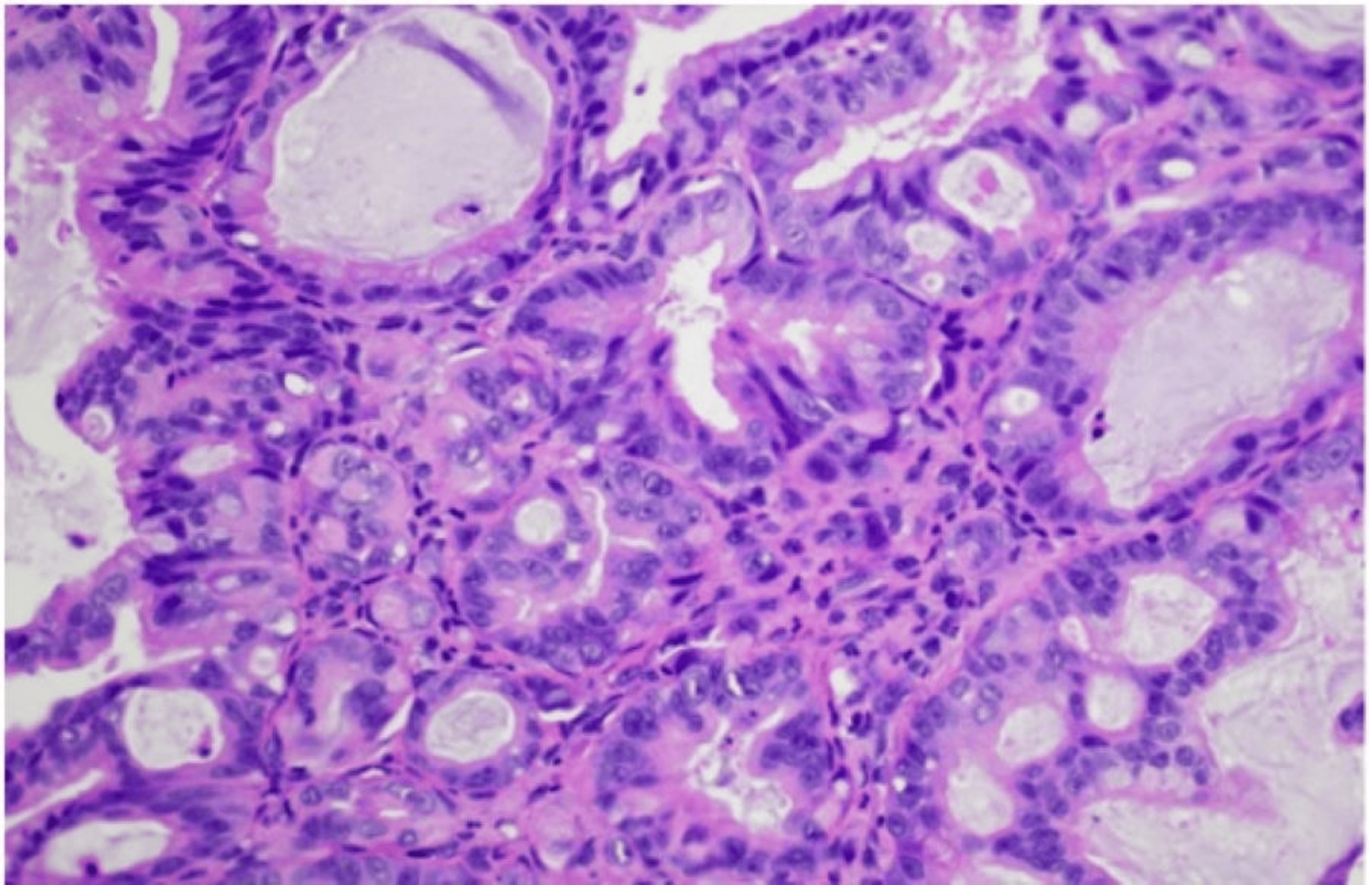
High power view (Hematoxylin & Eosin, 40x) The image highlights atypical gland-forming epithelial cells with hyperchromatic nuclei, prominent nucleoli, and mitotic activity, consistent with metastatic adenocarcinoma.

**Figure 4:**
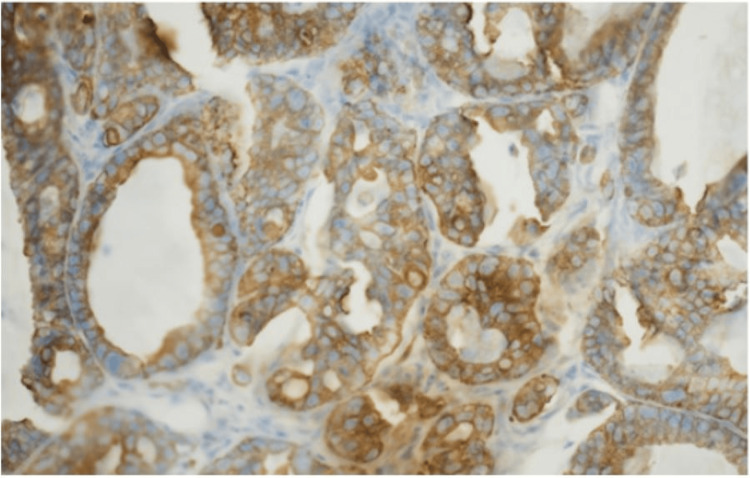
Pan-cytokeratin (AE1/AE3) immunostaining The image shows diffuse cytoplasmic positivity, confirming epithelial origin.

**Figure 5:**
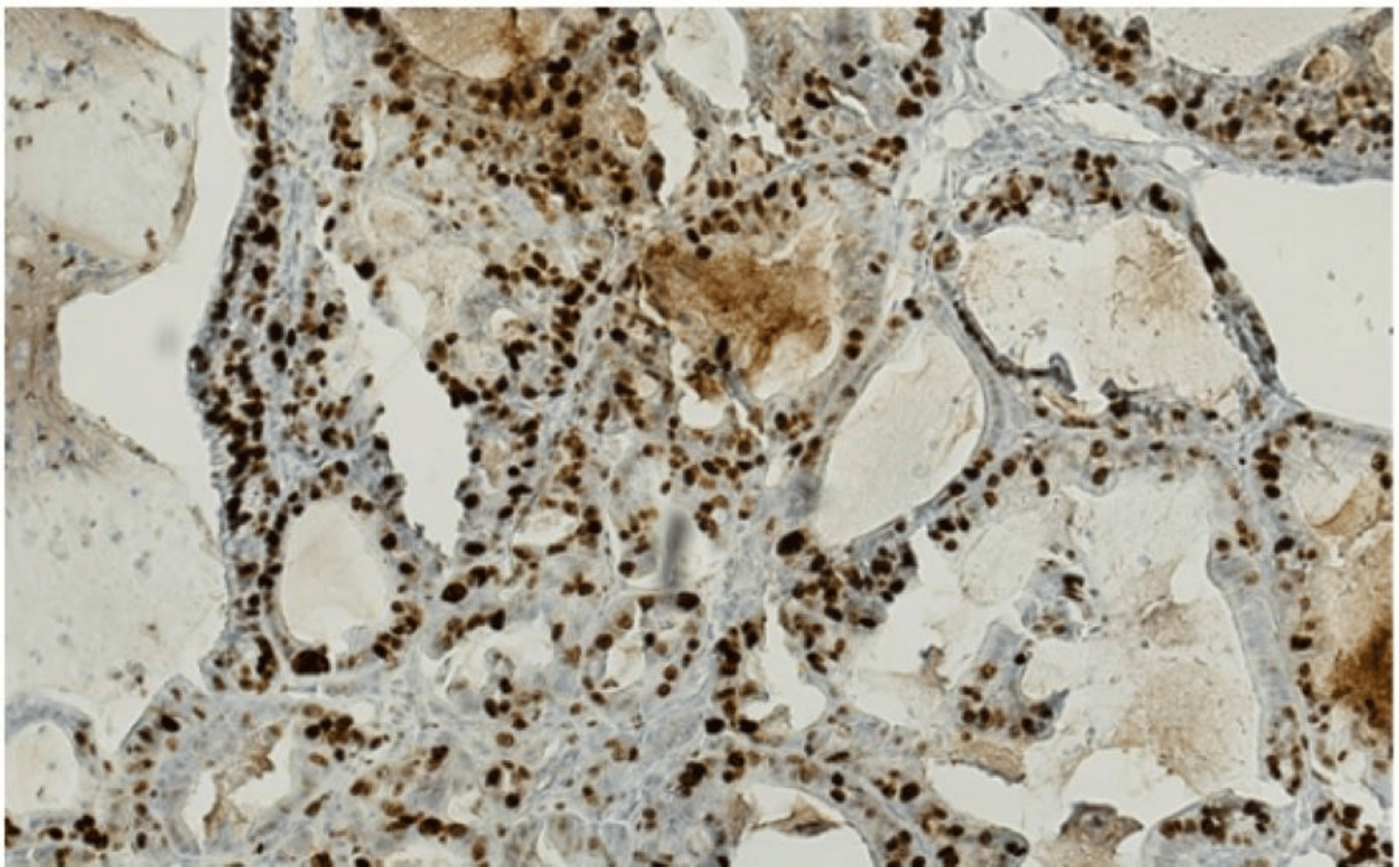
Ki-67 proliferation index The elevated index in tumor cells indicates high proliferation activity.

**Figure 6:**
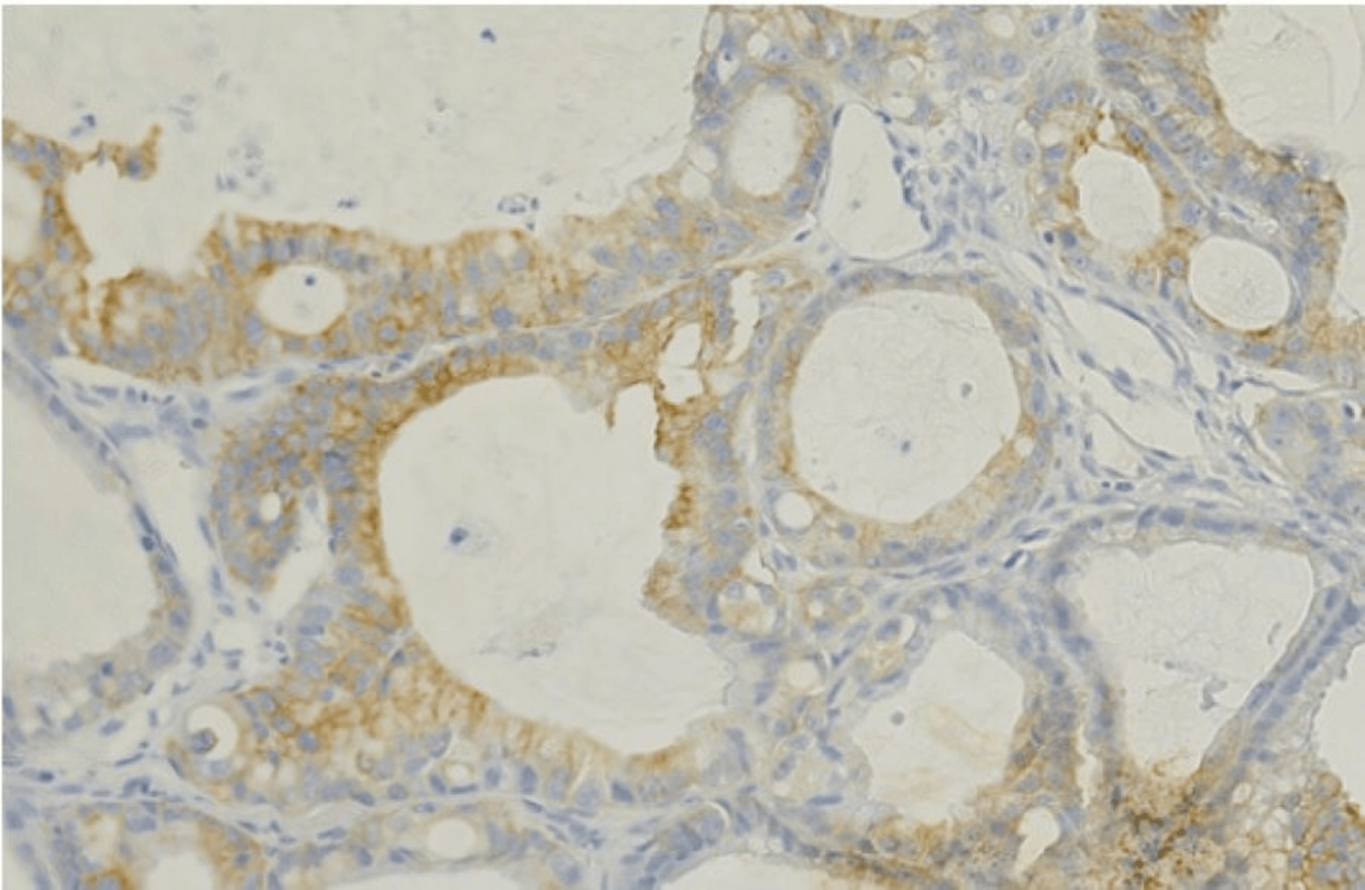
K7 stain The image shows weak cytoplasmic positivity, consistent with gastric origin.

Histological examination of the lip lesion revealed infiltrating irregular glandular structures within the dermis (Figure 3), composed of atypical epithelial cells with nuclear pleomorphism, hyperchromasia, and frequent mitoses. Immunohistochemical staining showed diffuse cytoplasmic positivity for pan-cytokeratin (AE1/AE3), confirming epithelial origin (Figure 4). The Ki-67 proliferation index was markedly elevated, indicating high proliferative activity (Figure 5). CK7 demonstrated weak cytoplasmic staining, supporting a gastric primary (Figure 6). CK20 and CD68 were negative. These morphologic and immunophenotyping findings support the diagnosis of metastatic adenocarcinoma, consistent with a known gastric primary. 

For his anemia, the peripheral smear showed normocytic normochromic anemia with mild polychromasia and a few target cells and rouleaux present. Hemoglobin improved from 6.5 to 8.5 following the transfusion of two units of PRBCs during the hospital course. 

He recovered well from his lesion excisions with a stable overall condition. Due to the patient’s continuing weight loss and anemia, increased risks of falls, and metastases, he was ineligible to recontinue chemotherapy unless he improved significantly. Oncology indicated his prognosis was poor.

## Discussion

Cutaneous metastasis is an uncommon manifestation of visceral malignancy, reported in only ~5% of patients with internal cancers, with pooled series showing a range of 0.7-9% [1,2].  Breast carcinoma is the single most frequent source, whereas lung, colorectal, renal, ovarian, and bladder primaries each account for roughly 3.4-4% of cases [3]. In contrast, metastases from the upper digestive tract are distinctly rare (< 1%) and gastric adenocarcinoma is responsible for barely 6% of all cutaneous deposits [4,5].  When gastric cancer does seed the skin, dissemination is usually limited to the abdominal wall, umbilicus, or scalp; widely distributed or acral lesions, such as the pedunculated nodules on our patient’s lower lip and chin, remain exceptional presentations [6,7].

Tumor cells reach the skin primarily via hematogenous or lymphatic routes, but direct extension and iatrogenic implantation have also been described [8]. Regardless of the pathway, the secondary cutaneous lesion typically reproduces the histology of the primary tumor.  In gastric adenocarcinoma, this often includes signet-ring morphology with intracytoplasmic mucin and eccentrically displaced nuclei, as was confirmed in both the lip and chin excisions of our patient [9].

The appearance of cutaneous metastasis generally marks disseminated disease and carries an adverse prognosis, especially for primaries in the lung, ovary, or upper gastrointestinal tract [10]. Management is therefore palliative: local excision, radiotherapy, or intralesional therapy is reserved for symptom control, whereas systemic chemotherapy is frequently ineffective once widespread cutaneous disease is evident [11].  In our patient, excision provided rapid relief of bleeding and discomfort, but profound anemia, cachexia, and probable pulmonary metastases precluded the resumption of cytotoxic therapy.

Early recognition remains crucial.  Persistent indurated erythema, rapidly enlarging nodules or papules of undetermined origin in any patient with current or prior malignancy merit prompt biopsy.  Timely histological confirmation can identify metastatic spread before crippling visceral disease develops, allowing clinicians to discuss prognosis candidly and institute symptom-directed care without delay.  This case reinforces that vigilance for atypical cutaneous manifestations is essential even in cancer patients with good follow-up.

## Conclusions

In summary, this case illustrates that although cutaneous metastasis from gastric adenocarcinoma is an exceptionally rare event, its appearance almost invariably signals advanced systemic spread and a grave prognosis. Rapidly evolving or indurated skin lesions, even at atypical sites such as the lip and chin, should therefore prompt urgent biopsy in patients with known or suspected malignancies. Early histopathologic confirmation not only clarifies the diagnosis but also allows clinicians to initiate timely, symptom-directed interventions and to counsel patients and families realistically about expected outcomes. While local excision can provide meaningful palliation, most patients with cutaneous dissemination are best served by a comprehensive, comfort-focused approach that prioritizes quality of life over aggressive systemic therapy when the latter is unlikely to alter the disease course. 
